# Tracheal schwannoma: A pseudo-asthmatic syndrome with successful laser Nd-YAG resection as first-line therapy

**DOI:** 10.1016/j.ijscr.2025.111706

**Published:** 2025-07-22

**Authors:** Ranim Nakhal, Raneem Ahmad, Bassel Ibrahim, Sultaneh Haddad, Arabi Abbas, Nizar Abbas

**Affiliations:** a Department of Pathology, Faculty of Medicine, Damascus University, Damascus, Syria; b Stemosis for Scientific Research, Damascus, Syria; c Department of Thoracic Surgery, The National University Hospital, Damascus, Syria; d Department of Pediatric, Children's University Hospital, Damascus, Syria; e Damascus University, Faculty Of Medicine, Damascus, Syria

**Keywords:** Showannoma, Trachea, Nd-YAG laser

## Abstract

**Introduction:**

Neurogenic tumors like schwannoma are sporadic in less than 0.5 % of primary tracheal tumors (PTT). Schwannoma can be misdiagnosed with asthma because of nonspecific symptoms such as wheezing, coughing, or dyspnea.

**Case presentation:**

A 39-year-old woman presents to the emergency department with severe dyspnea at rest and needs oxygenation. Rigid Bronchoscopy revealed an intratracheal white pedunculated lesion with visible vessels. A frozen section's result was benign Showannoma. Nd-YAG Laser was performed to melt the tumor, and then the lesion was swept away. After that, the Nd-YAG laser was used to remove tumor remnants.

**Discussion:**

Our case emphasizes that rigid bronchoscopy with Nd-YAG laser resection was a very successful tool for managing schwannoma, and bronchoscopic surveillance showed No Recurrence as the benign nature of pedunculated schwannoma without an extratracheal component.

**Conclusion:**

Awareness of the possibility of schwannoma as a primary tracheal tumor is important, although it is phenomenal, it can cause pseudo-asthmatic status because it has non-specific symptoms.

## Highlights

• Neurogenic tumors like schwannoma are sporadic for less than 0.5 % of primary tracheal tumors (PTT)
• Nd-YAG Laser was performed to melt the tumor, and then the lesion was swept away. After that, the Nd-YAG laser was used to remove tumor remnants.

## 1 Introduction

PTT depicts 2 % of upper airway tumors and 0.1–0.4 % of all malignancies. Moreover, 90 % of them are malignant in adults [1,2]. Neurogenic tumors like schwannoma and neurofibromas are sporadic in less than 0.5 % of PTT, and 0.2 % are for schwannoma [1,[3], [4], [5]]. Clinically, schwannoma can be misdiagnosed with asthma because of nonspecific symptoms such as wheezing, coughing, or dyspnea. The response to bronchodilators or corticosteroids is characteristic of asthma. Failure to observe an improvement in lung function should prompt the consideration of other investigative methods to exclude pseudo-asthmatic syndromes. [[6], [7], [8]] Laser resection has been used increasingly over the last decade without significant morbidity, and the Nd-YAG laser should be considered first-line therapy in benign endobronchial tumors within certain conditions [9,10]. In our case, we presented a schwannoma tumor that made a pseudo-asthmatic syndrome, emphasizing that rigid bronchoscopy with Nd-YAG laser resection was a very successful tool for managing schwannoma, and bronchoscopic surveillance showed no recurrence.

## 2 Case presentation

A 39-year-old female presented to the emergency department with a two-year history of progressive dyspnea and a persistent dry cough. The symptoms had gradually worsened over time, prompting medical evaluation. During that period, she was treated as an asthmatic patient with bronchodilators and steroids with little improvement. Additionally, her respiratory symptoms were significantly exacerbated during pregnancy, necessitating frequent medical interventions for recurrent respiratory infections and hypoxemia, eventually leading to oxygen dependence. Following childbirth, she required urgent hospitalization.

**Fig. 1:**
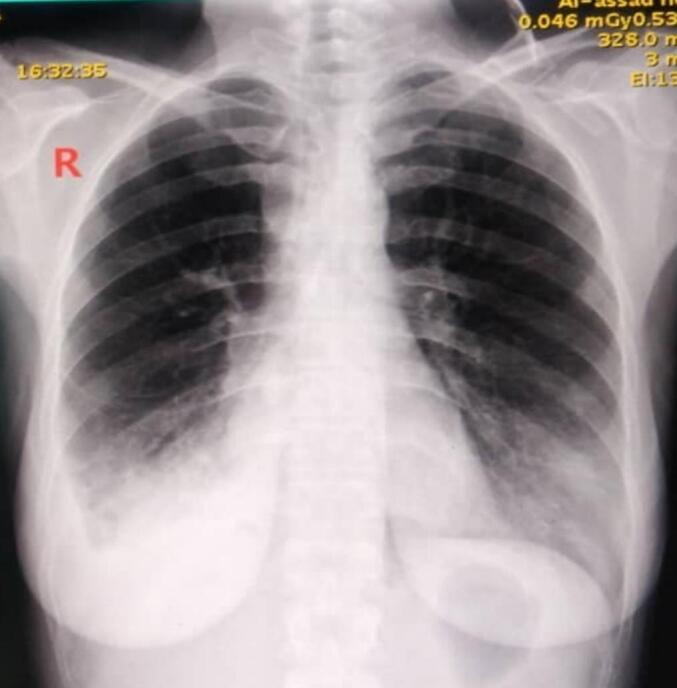
Chest x-ray showing A shadow in the trachea with bilateral pulmonary infiltrates and right angle closure

**Fig. 2:**
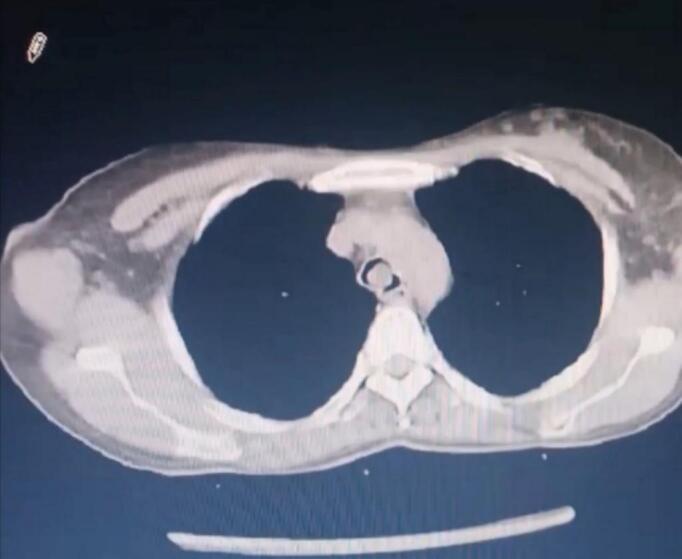
CT scan showing a large lesion obstructing 95 % of the inferior third of the tracheal lumen without extratracheal component.

**Fig. 3:**
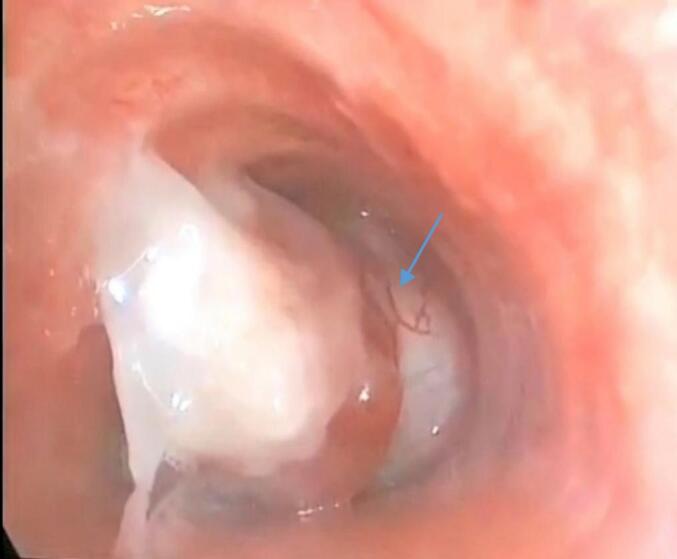
Bronchoscopic view. An intratracheal white pedunculated lesion with visible vessels (the arrow).

Clinical examination revealed the presence of stridor. Vital signs were as follows: blood pressure 120/70 mmHg, pulse rate 88 beats per minute, body temperature 37 °C, respiratory rate 28 breaths per minute, and oxygen saturation 85 % on room air. The other systems examination showed no additional abnormalities. A shadow in the trachea with bilateral pulmonary infiltrates and right angle closure was on the chest X-ray [Fig. 1]. CT-Scan showed a large lesion obstructing 95 % of the inferior third of the tracheal lumen without extratracheal component [Fig. 2]. Rigid Bronchoscopy revealed an intratracheal white pedunculated lesion with visible vessels, occupying 95 % of the lumen [Fig. 3]. A frozen section's result was benign Showannoma. Nd-YAG Laser was performed to melt the tumor, and then the lesion was removed by large biopsy forceps. After that, the Nd-YAG laser was used to coagulate and remove the tumor remnant. The procedure was performed under general anesthesia and lasted approximately 30 min.

**Fig. 4:**
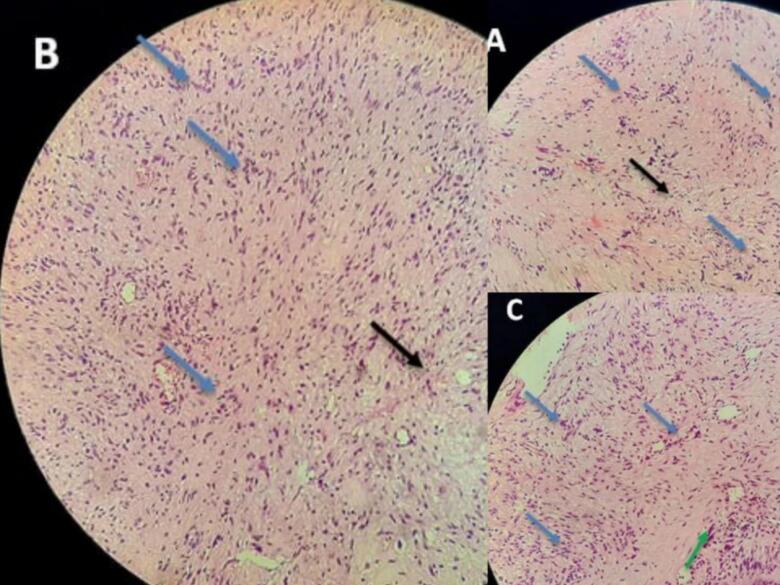
Hematoxylin and eosin stain: Spindle cells with twisted nuclei with indistinct cytoplasmic borders, Antoni A areas with nuclear palisading (blue arrows), Antoni B areas (black arrows), and squamous metaplasia with low-grade dysplasia in the tracheal epithelium (green arrow).

**Fig. 5:**
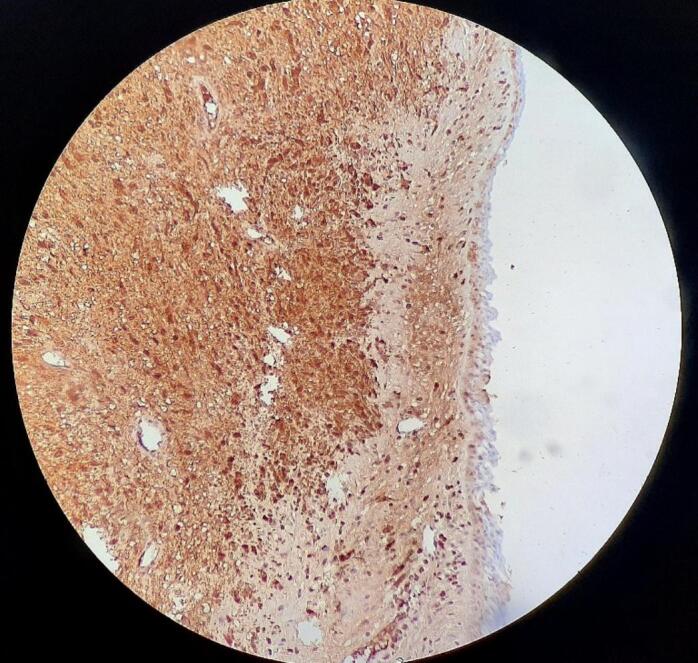
S-100 protein was positive on immunohistochemical test.

**Fig. 6:**
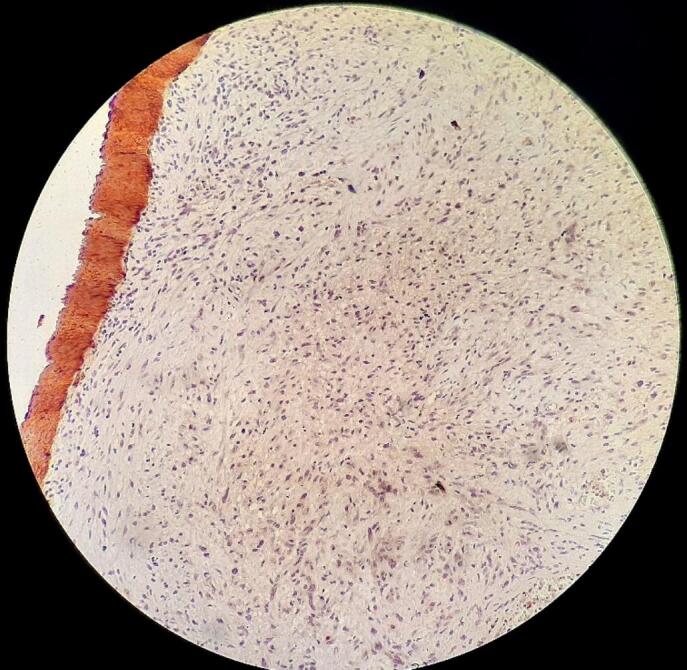
CK stain was negative on the immunohistochemical test.

A routine pathologic study consistent with schwannoma revealed Spindle cells in short fascicles, hypercellular areas (Antoni A) with nuclear palisading, paucicellular areas (Antoni B), and squamous metaplasia with low-grade dysplasia in the tracheal epithelium [Fig. 4]. S-100 protein immunoreactivity was positive [Fig. 5], and CK stain (Cytokeratin AE1/AE3) was negative [Fig. 6], confirming the schwannoma diagnosis.

No recurrence was observed through follow-up by flexible bronchoscopy at 10 months postoperatively.

## 3 Discussion

Primary Tracheal Tumors (PTT) depict 2 % of upper airway tumors and 0.1–0.4 % of all malignancies. Moreover, 90 % of them are malignant in adults [1,2].

PTT can originate from epithelial, adenoid, or mesenchymal sources. Two-thirds (85 %) of adults' PTT consists of squamous and adenoid cystic carcinoma, with the same incidence rate [1,2,11].

Benign tumors, low-grade malignancies, and other wide variety of histological subtypes make up the last third. Thus, Neurogenic tumors like schwannoma and neurofibromas are sporadic in less than 0.5 % of PTT [1,3,4].

Schwannoma is a well-circumscribed encapsulated neoplasm, connected to the nerve sheath loosely without invading it, with sporadic occurrence, while a small percentage is associated with von Recklinghausen's disease [12,13]. The most common locations for it are the cerebellopontine angle, flexor surfaces of the extremities, mediastinum, neck, and posterior spinal roots [12,13]. Malignant transformation of schwannoma is exceptionally rare and mostly exhibits an epithelioid morphology [13].

Tracheal schwannoma is an uncommon presentation, most common in the distal bronchial tree or lung parenchyma, with a female gender preference for a male-to-female ratio of 7:21. [7,[14], [15], [16], [17]] The distal third of the trachea is more involved, followed by the proximal and then the middle third [17].

Diagnosis can be challenging, with an average delay of 17 months from the onset of symptoms [17]. Thus, some points need to be highlighted to avoid delay and misdiagnosis:

• Symptoms appear as non-specific clinical manifestations when the tumor occludes around 50–70 % of the tracheal lumen, while 30–75 % of the chest radiograph shows normal findings [1,18].
• Symptoms can mimic asthma and create a pseudo-asthma status:

Cough, wheezing, and dyspnea result from causes other than asthma [2,6,8]. Most chronic cough cases in adults are caused by asthma (the most common etiology), gastroesophageal reflux disease, and chronic rhinosinusitis. Thus, it is a presentation of nonpulmonary disorders as well as pulmonary ones [19]. Moreover, stridor or wheezing can be mistaken for asthma, while it may arise in tracheal obstruction, foreign body, bronchomalacia, or even as a physician misestimating various respiratory sounds [1,6,8,20].

Substantial improvement of airway obstruction from an aerosol bronchodilator (greater than 12 % and a greater than 250 mL increase in the forced expiratory volume in 1 s (FEV 1) after bronchodilator) or a short course of reasonably high-dose systemic corticosteroid, 2 mg/kg twice daily to a maximum of 40 mg twice daily, is characteristic of diagnosing asthma. Failure to observe a relief and improvement in lung function within 5 to 7 days or 10 days maximum, should consider a fiberoptic bronchoscopy or CT scan to evaluate the airways for any other causes as the etiology. [[6], [7], [8]] Pseudo-asthmatic syndromes are shown in [Table 1]. [8]

**Table 1:** Pseudo-asthmatic syndromes.

Bronchial inflammatory diseases
Infectious asthma
Bronchiolitis and croup
Chronic asthmatic bronchitis
Cystic fibrosis
Emphysema
Central airway obstruction
Cardiac asthma
Aspiration syndromes
Pulmonary embolism
Carcinoid syndromes
Hypersensitivity pneumonitis
Pulmonary infiltration with eosinophilia
Sarcoidosis
Psychogenic dyspnea and factitious asthma

Central airway obstruction (CAO) as a Pseudo-asthmatic syndrome can result from a wide variety of diseases shown in [Table 2]. [8]

**Table 2:** Important causes of central airway obstruction.

Obstruction Type	Characteristics
Pharyngeal	Retropharyngeal abscess
	Ludwig's angina
	Tonsil or adenoid enlargement
Laryngeal	Vocal cord palsy
	Epiglottitis Laryngeal edema
	Tumor, polyp
	Relapsing polychondritis
Extrinsic trachea/bronchial compression	Mediastinal tumor
	Aneurysm
	Mediastinal abscess
	Goiter
Intramural tracheal/bronchial disease	Stricture
	Fracture
	Tracheomalacia
	Tracheopathia osteoplastica
	Relapsing polychondritis
	Wegener's granulomatosis
Intraluminal tracheal disease	Tumors, benign or malignant
	Tracheitis dessicans
	Relapsing polychondritis
	Foreign body
Nonorganic upper airway	Functional syndromes obstruction
Benign central airway stenosis	Lung transplantation
	Infection
	Congenital lesions
	Tracheobronchial malacia
	Acquired immunodeficiency syndrome
	External compression from benign
	mediastinal masses or fibrosis

One or more of the following diagnostic maneuvers should be used if one or more previously mentioned clinical features appear and suggest a CAO [8]:

• The standard chest roentgenograms with careful inspection of tracheal air shadow.
• Tracheal tomograms.
• CT scan of neck or chest with three-dimensional reconstruction.
• Laryngoscopy.
• Bronchoscopy.
• Mediastinoscopy or mediastinotomy.

In our patient, chest roentgenograms and CT scans were the key to diagnosing the tumor. Then, an emergency bronchoscopy was performed.

A CT scan is the most useful radiological way to estimate tracheal tumors (size, site, extratracheal extension). However, bronchoscopy is the most important diagnostic tool for taking crucial information about the trachea, tumor location, and dimensions. Moreover, bronchoscopy allows us to take biopsies for tumor identification and to plan for surgical resection in consideration of all reservations that are needed in suspected CAO [1].

The endoscopic appearance of schwannoma can be well-circumscribed, round, raised, or smooth gray masses, and visible vessels or cystic areas can exist [9,12,13]. However, only microscopic characters can give an accurate diagnosis; typical dense and loose areas known as Antoni A and Antoni B areas respectively, Verocay bodies which are formed by central nuclear-free zones ramified by palisading nuclei, a characteristic spindled elongated nucleus with a wavy of buckled shape, thick-walled hyalinized blood vessels, axons absence, cystic changes, and S100 positivity, calretinin, basal lamina component (such as laminin and type IV collagen) as immunohistochemical features [12,13]. Hence, pathologic spindled cells can be excluded [Table 3]. [12,13,21]

**Table 3:** The key histological differential diagnoses (DD) for schwannoma.

Diagnosis	Histological Features	Immunohistochemistry (IHC)	Key Distinguishing Features
Neurofibroma	Loosely arranged spindle cells, wavy nuclei, myxoid background, unencapsulated, lacks Antoni A/B and Verocay bodies.	S-100 positive (less diffuse than schwannoma)	Not encapsulated, lacks biphasic pattern
MPNST (Malignant Peripheral Nerve Sheath Tumor)	Highly cellular, atypia, high mitoses, necrosis, infiltrative growth Spindeled cells arranged in sweeping fascicles with variations in cellularity	S-100 (focal/variable), SOX10 (+), H3K27me3 loss (often)	Malignant features, patchy S-100, unencapsulated
Leiomyoma/Leiomyosarcoma	Intersecting fascicles of spindle cells with eosinophilic cytoplasm and blunt-ended nuclei, increased mitoses, and necrosis in sarcoma	SMA positive, Desmin positive, S-100 negative	Smooth muscle markers positive, different nuclear morphology
Fibrosarcoma	Malignant spindle cells in herringbone pattern, high mitotic rate	Vimentin positive, S-100 negative	Herringbone architecture, S-100 negative
Solitary Fibrous Tumor (SFT)	Patternless pattern of spindle cells, prominent branching (“staghorn”) vessels	CD34 positive, CD99 positive, STAT6 nuclear positive, S-100 negative	STAT6 nuclear positivity is diagnostic
Spindle Cell Melanoma	Spindle cells may mimic schwannoma, sometimes pigmented	S-100 positive, HMB-45 positive, Melan-A positive, SOX10 positive	Melanocytic markers (HMB-45, Melan-A) distinguish it
Inflammatory Myofibroblastic Tumor (IMT)	Spindle cells in myxoid or collagenous stroma, mixed inflammatory infiltrate	ALK positive (often), SMA positive, S-100 negative	Inflammatory background, ALK+, no Antoni/Verocay bodies
Meningioma	Whorled cell pattern, psammoma bodies, with intersecting fascicles of spindled cells	EMA positive, S-100 negative, or focal	Whorls and psammoma bodies, EMA+, lack encapsulation

**Fig. 7:**
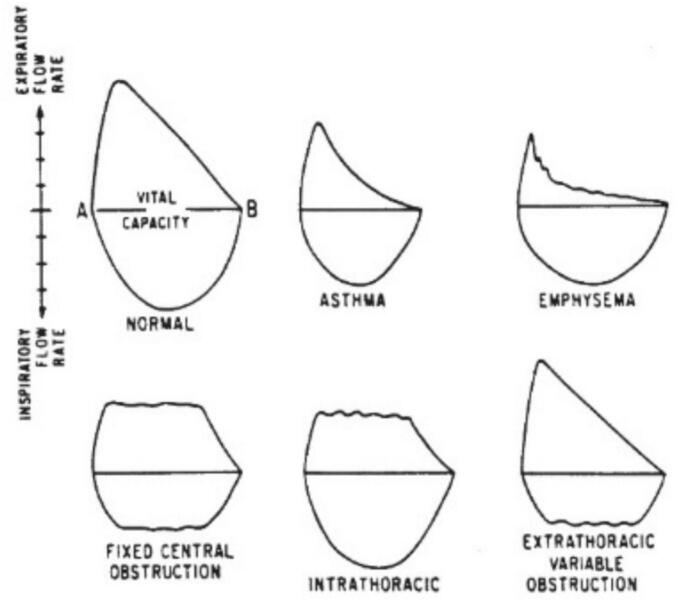
Flow volume loops; flattening of the inspiratory limb in extrathoracic airflow obstruction; flattening of the expiratory limb in intrathoracic airflow obstruction; obvious reduction in inspiratory and expiratory flows in asthma.

It is noteworthy to highlight other useful steps for forming an early diagnosis to exclude confusion between asthma and CAO: PFTs as an essential step, Spirometry (sensitive for quantifying airway obstruction), and Flow-volume loop (more beneficial in CAO recognition) [Fig. 7] [8,22]. The emergency status of our patient was unable to perform pulmonary function testing.

The appropriate choice of treatment should consider the risk of tracheal resection, the tumor's clinical findings, size, shape (pedunculated or sessile), whether an extratracheal component is present, or whether it is a completely intraluminal tumor, as well as the patient's cardiopulmonary function [17,23].

The standard treatment of benign PTT and intermediate aggressiveness is surgical resection with reconstruction of the airway, which is also the optimal procedure for patients with huge and sessile schwannomas [1,17,24]. Other methods to treat tracheal schwannoma include electrocautery snaring, argon plasma coagulation, cryotherapy, endoscopic excision, and microdebridement [Table 4] [17,25]. In addition, an endoscopic laser has successfully managed benign PTT, like schwannoma, and it is what we used in our case, combined with large biopsy forceps, as it was the best option because of the large tumor (95 %), tracheal obstruction, and high vascularity. [10,26,27]

**Table 4:** Other methods to treat tracheal schwannoma.

	Technique Laser	Electrocautery	Mechanical Debulking	Cryotherapy
Mechanism	Dehydration Deep coagulation	Superficial cauterization. Cutting.	Physical removal using forceps.	Destroy tissue by freezing.
Efficacy in tumors	Vascular/deep tumors	Small/medium lesions.	After laser or electrocautery to remove the remains	Adjunct for devitalized tissue
Speed of action	Fast	Fast to moderate	Depends on tumor size and texture	Slow(may require multiple sessions)
Bleeding risk	Low	Moderate	Moderate to high if used alone.	Low
Risk of airway fire	High (with O2 > 40 %)	High	None	None
The best role in multimodal management	Vascular tumors	Soft/mucoid lesions	Used to clean/remain patent	Post-thermal for healing

Laser resection has been used increasingly over the last decade without significant morbidity, and Nd-YAG laser should be considered first-line therapy in benign endobronchial tumors within certain conditions: strictly endoluminal, low recurrence possibility, the limited extent within the endobronchial tree, recent lobe/lung collapse, symptomatic airway compromise, and poor surgical risks [9,10]. Prognosis outcomes of different types of lasers are shown in [Table 5] [28]. Bronchoscopic surveillance is highly recommended for remote recurrence if the resection is incomplete. Thus, surgery should be chosen for local recurrence [9,10,17].

**Table 5:** Prognosis outcomes of different types of lasers.

	Nd:YAG Laser	CO2 Laser	Diode Laser:
Benign pedunculated neoplasms	-Deep breakthrough. -Ideal.	-Very accurate. -Minimal thermal damage. -Surface cutting.	_
Benign sessile neoplasms	In case of deeper tissue impact is needed (the used care).	-Minimal damage. -Superficial ablation.	_
Malignant neoplasms	-First-line. -Good coagulation. -Palliative debulking.	Superficial only.	Similar coagulation to Nd:YAG.

Using rigid equipment enhances the surgeon's ability to remove a tumor more rapidly in combination with laser coagulation. The flexible bronchoscope is then passed through the rigid resection scope to permit the use of the Nd: YAG laser to complete the debridement and obtain hemostasis [29].

Our case emphasizes that rigid bronchoscopy with Nd-YAG laser resection was a very successful tool for managing schwannoma, and Bronchoscopic surveillance showed No Recurrence as the benign nature of the schwannoma without an extratracheal component. The work has been reported in line with the SCARE criteria [30].

## 4 Conclusion

Awareness of the possibility of schwannoma as a primary tracheal tumor is important, although it is phenomenal, it can make pseudo-asthmatic status because it has non-specific symptoms (persistent cough, whizzing, dyspnea) that are not relieved by bronchodilators and may cause CAO. Therefore, Spirometry, PTFs, CT, and bronchoscopy should be performed to make the correct diagnosis. Treatment choice depends on the tumor's clinical findings, size, shape (pedunculated or sessile), extratracheal component, and the patient's condition. Skilled recognition of the endoscopic appearance is necessary. Rigid bronchoscopy with Nd-YAG laser resection is a successful tool for managing schwannoma. However, long-term follow-up after tumor resection is required.

## CRediT authorship contribution statement

**RN** contributed to drafting, reviewing, editing and approved the final manuscript.

**RA** contributed to drafting, reviewing, editing and approved the final manuscript.

**BI** contributed to drafting, reviewing, editing and approved the final manuscript.

**SH**contributed to drafting, editing and approved the final manuscript.

**AA** contributed to drafting, reviewing, editing, corresponding, and approved the final manuscript.

**NA** contributed to reviewing, supervising and approved the final manuscript.

## Consent

Written informed consent was obtained from the patient for publication and any accompanying images. A copy of the written consent is available for review by the Editor-in-Chief of this journal on request.

## Ethical approval

Ethical approval is not required for this type of publication.

## Guarantor

All authors have read and approved the manuscript, on behalf of all the contributors I will act and guarantor and will correspond with the journal from this point onward.

## Sources of funding

Not applicable.

## Declaration of competing interest

Not applicable.

## References

[1] Sammartino F., Macchiarini P. (2014). Lung Cancer.

[2] Al-Qadi M.O., Artenstein A.W., Braman S.S. (2013). The “forgotten zone”: Acquired disorders of the trachea in adults. Respir. Med..

[3] Hamdan A.L., Moukarbel R.V., Tawil A., El-Khatib M., Hadi U. (2010). Tracheal schwannoma: a misleading entity. Middle East J. Anaesthesiol..

[4] Xu L.-T. (1987). Clinical and pathologic characteristics in patients with tracheobronchial tumor: report of 50 patients. Ann. Thorac. Surg..

[5] Lina G. (2023). Tracheobronchial schwannoma: a case report and literature review. J. Int. Med. Res..

[6] Weinberger M., Abu-Hasan M. (2007). Pseudo-asthma: when cough, wheezing, and dyspnea are not asthma. Pediatrics.

[7] Righini C.A., Lequeux T., Laverierre M.-H., Reyt E. (2005). Primary tracheal schwannoma: one case report and a literature review. Eur. Arch. Otorhinolaryngol..

[8] Lillington G.A., Faul J.L. (2001). Bronchial Asthma.

[9] Shah H. (1995). Benign tumors of the tracheobronchial tree. Chest.

[10] Jung Y.Y. (2013). Bronchial schwannomas: clinicopathologic analysis of 7 cases. Korean J Pathol.

[11] Onal M., Ernam D., Atikcan S., Memiş L. (2009). Endobronchial schwannoma with massive hemoptysis. Tuberk. Toraks.

[12] Hoda S.A., Cheng E. (2017). Robbins basic pathology. Am. J. Clin. Pathol..

[13] Rosai J. (2011).

[14] Shigematsu H., Aoe M., Date H. (2007). Schwannoma occurring from the lingular bronchus. Eur. J. Cardiothorac. Surg..

[15] Miller D.R. (1969). Benign tumors of lung and tracheobronchial tree. Ann. Thorac. Surg..

[16] Pang L.-C. (1989). Primary neurilemoma of the trachea. South. Med. J..

[17] Ge X., Han F., Guan W., Sun J., Guo X. (2015). Optimal treatment for primary benign intratracheal schwannoma: a case report and review of the literature. Oncol. Lett..

[18] Grillo H.C., Mathisen D.J. (1990). Primary tracheal tumors: treatment and results. Ann. Thorac. Surg..

[19] Linz A.J. (2007). The relationship between psychogenic cough and the diagnosis and misdiagnosis of asthma: a review. J. Asthma.

[20] Cane R.S. (2000). What do parents of wheezy children understand by ‘wheeze’?. Arch. Dis. Child..

[21] Reddy V.B., David O., Spitz D.J., Haber M.H. (2021).

[22] Crapo R.O. (1994). Pulmonary-function testing. N. Engl. J. Med..

[23] Rusch V.W., Schmidt R.A. (1994). Tracheal schwannoma: management by endoscopic laser resection. Thorax.

[24] Grillo H.C., Mathisen D.J. (1990). Primary tracheal tumors: treatment and results. Ann. Thorac. Surg..

[25] Guibert N. (2016). Techniques of endoscopic airway tumor treatment. J. Thorac. Dis..

[26] Dincer S.I., Demir A., Kara H.V., Fener N., Altin S. (2006). Primary tracheal schwannoma: a case report. Acta Chir. Belg..

[27] Rusch V.W., Schmidt R.A. (1994). Tracheal schwannoma: management by endoscopic laser resection. Thorax.

[28] Grillo H.C. (2004).

[29] Patterson G.A. (2008).

[30] Kerwan A., Al-Jabir A., Mathew G., Sohrabi C., Rashid R., Franchi T., Nicola M., Agha M., Agha R.A. (2025). Revised surgical CAse REport (SCARE) guideline: an update for the age of artificial intelligence. Premier Journal of Science.

